# Extremity Necrosis Due to Intrauterine Arterial Ischemia

**DOI:** 10.4274/tjh.galenos.2020.2020.0530

**Published:** 2021-08-25

**Authors:** Serdar Beken, Kerim Sarıyılmaz, Eda Albayrak, Arzu Akçay, Ayşe Korkmaz

**Affiliations:** 1 Acıbadem University Faculty of Medicine, Department of Pediatrics, Neonatology Subdivision, İstanbul, Turkey; 2 Acıbadem University Faculty of Medicine, Department of Orthopedics, İstanbul, Turkey; 3 Acıbadem University Faculty of Medicine, Department of Pediatrics, İstanbul, Turkey; 4 Acıbadem University Faculty of Medicine, Department of Pediatrics, Pediatric Hematology Subdivision, İstanbul, Turkey

**Keywords:** Newborn, Ischemia, Arterial

A preterm male infant weighing 1230 g was delivered by cesarean section at 32^1/7^ gestational weeks. His twin weighed 1300 g and neither required resuscitation. On physical examination, the left lower extremity was rudimentary, pale, shorter than the right, and had a club-foot deformity. Computerized tomographic angiography demonstrated a bluntly filled femoral artery with no distal passage. During follow-up, the demarcation line became clear on the 5^th^ day and amputation was performed under the left hip joint (Figure 1).

Laboratory tests including homocysteine, folate, prothrombin time, activated partial thromboplastin time, protein C and S activity, fibrinogen, and antithrombin III activity were within normal limits according to gestational age, except for high D-dimer (4.13 mg/dL). Antiphospholipid and anticardiolipin antibodies were also negative. The patient was found to be homozygous for methylenetetrahydrofolate reductase C677T; thrombosis was not observed in histopathological examination. The patient was successfully discharged from the hospital on the 40^th^ day of life.

Arterial ischemia of the limb can be seen after thromboembolic events or vascular interventions [1,2]. Ischemic changes present at birth suggested intrauterine onset causes that might be attributable to occlusive vascular disruption [3]. Diminished blood flow altered normal soft tissue and osseous growth and resulted in ischemia in fetal life, ending with limb loss.

## Figures and Tables

**Figure 1:**
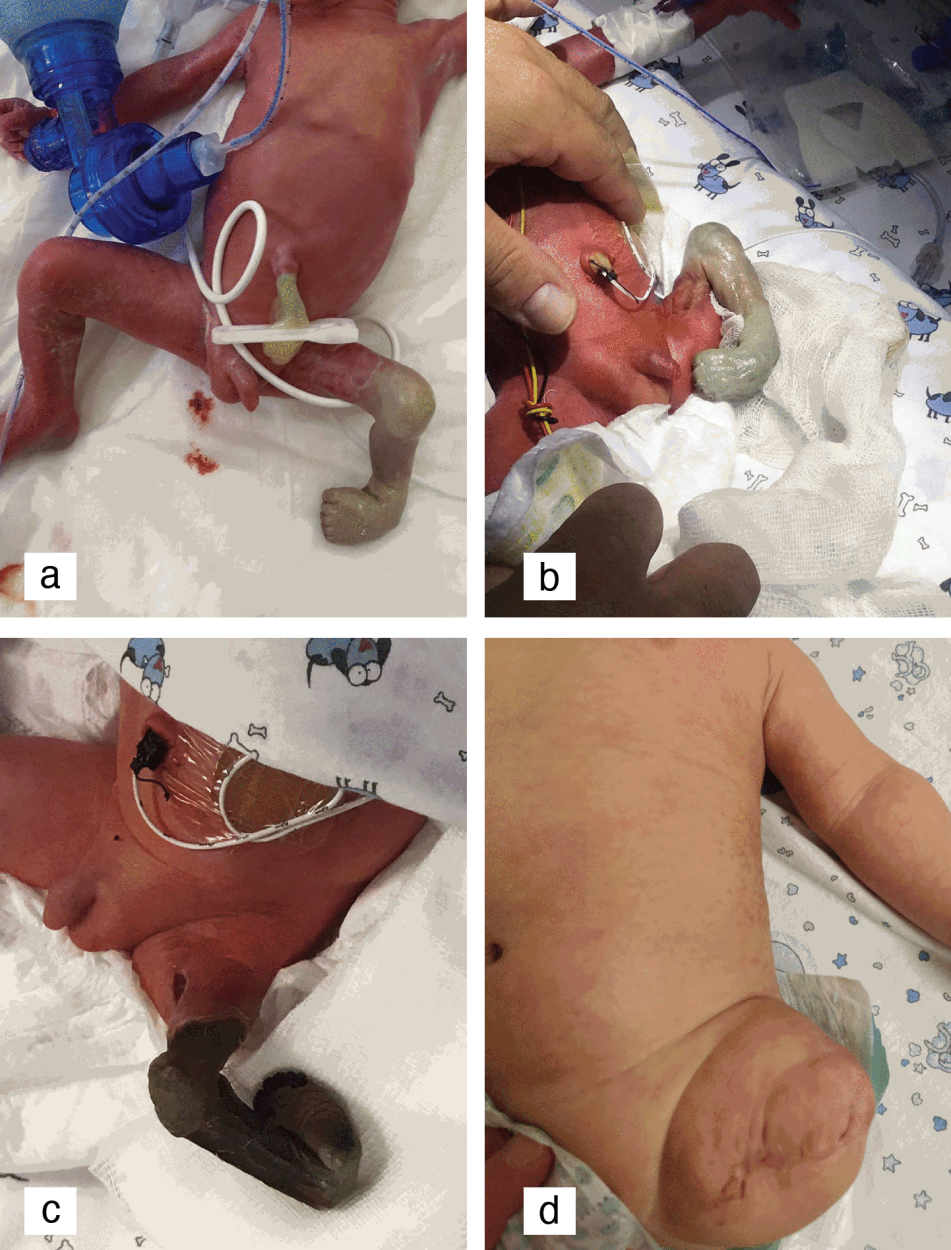
(a-d) Left lower extremity was rudimentary, pale, shorter than the right, and had a club-foot deformity. The demarcation line became clear on the 5^th^ day and amputation was performed under the left hip joint.
